# Age-Stratified Classification of Common Middle Ear Pathologies Using Pressure-Less Acoustic Immittance (PLAI™) and Machine Learning

**DOI:** 10.3390/healthcare13151921

**Published:** 2025-08-06

**Authors:** Aleksandar Miladinović, Francesco Bassi, Miloš Ajčević, Agostino Accardo

**Affiliations:** 1 Institute for Maternal and Child Health-IRCCS “Burlo Garofolo”, 34137 Trieste, Italy; 2 Department of Engineering and Architecture, University of Trieste, via A. Valerio 10, 34127 Trieste, Italy; francesco.bassi@phd.units.it (F.B.); majcevic@units.it (M.A.); accardo@units.it (A.A.)

**Keywords:** Pressure-Less Acoustic Immittance (PLAI), middle ear diagnostics, pediatric audiology, age-specific classification, Otitis Media, false negatives, ear canal acoustics

## Abstract

Background/Objective: This study explores a novel approach for diagnosing common middle ear pathologies using Pressure-Less Acoustic Immittance (PLAI™), a non-invasive alternative to conventional tympanometry. Methods: A total of 516 ear measurements were collected and stratified into three age groups: 0–3, 3–12, and 12+ years, reflecting key developmental stages. PLAI™-derived acoustic parameters, including resonant frequency, peak admittance, canal volume, and resonance peak frequency boundaries, were analyzed using Random Forest classifiers, with SMOTE addressing class imbalance and SHAP values assessing feature importance. Results: Age-specific models demonstrated superior diagnostic accuracy compared to non-stratified approaches, with macro F1-scores of 0.79, 0.84, and 0.78, respectively. Resonant frequency, ear canal volume, and peak admittance consistently emerged as the most informative features. Notably, age-based stratification significantly reduced false negative rates for conditions such as Otitis Media with Effusion and tympanic membrane retractions, enhancing clinical reliability. These results underscore the relevance of age-aware modeling in pediatric audiology and validate PLAI™ as a promising tool for early, pressure-free middle ear diagnostics. Conclusions: While further validation on larger, balanced cohorts is recommended, this study supports the integration of machine learning and acoustic immittance into more accurate, developmentally informed screening frameworks.

## 1. Introduction

The current landscape of general population otologic screening highlights a critical need for early detection and intervention strategies for hearing loss and related otologic conditions [1]. Early assessments of children’s hearing and periodic screenings throughout life are essential for identifying otologic diseases, thereby mitigating the health and social consequences associated with hearing impairment [2,3,4]. The World Health Organization (WHO) estimates that over 360 million individuals globally experience disabling hearing loss, emphasizing the urgency for effective screening strategies, particularly in low- and middle-income countries where healthcare access is often limited [5]. In pediatric populations, otologic screening is increasingly recognized as vital due to the high incidence of conditions such as acute otitis media, which affects over 80% of children by age three [6].

The American Academy of Audiology’s Clinical Practice Guidelines for Childhood Hearing Screening recommend initial assessments using pure tone audiometry, followed by tympanometry [7]. If pure tone assessment is not feasible, otoacoustic emission measurements are employed [8]. This structured approach underscores the necessity for comprehensive screening protocols that address both hearing and developmental concerns, particularly since untreated hearing loss can lead to significant cognitive and educational deficits [9,10,11,12].

In contrast, otologic screening for adults, especially those aged 50 and older, remains inconsistent. The U.S. Preventive Services Task Force (USPSTF) has historically stated that there is insufficient evidence to recommend routine screening for hearing loss in asymptomatic adults, which has resulted in a lack of standardized practices in primary care settings [13,14,15]. However, recent studies advocate for the integration of hearing screening into routine health assessments, emphasizing that early detection can significantly improve quality of life and cognitive outcomes [16,17]. Hearing loss has been shown to severely affect social function and mental health, contributing to cognitive decline and isolation in both pediatric and adult populations [8,18,19].

While screening programs are well established among children and industrial workers exposed to occupational hazards, broader population-level implementation remains limited. Disparities in screening rates, particularly among older adults and socioeconomically disadvantaged groups, underline the need for scalable, low-cost, and effective diagnostic technologies.

A key limitation in current screening practice lies in the diagnostic methods themselves. Tympanometry remains a gold standard due to its low cost and ease of use, and it is supported by robust clinical literature. It measures ear function via changes in acoustic impedance at a single frequency under variable air pressure [20]. However, the method is not without drawbacks: it can induce discomfort or even risk damage in sensitive patients [21], and may produce similar readings across diverse pathologies [22]. Efforts to improve diagnostic resolution through wideband acoustic immittance (WAI), which extends the frequency range of analysis, have shown promise, but the complexity of data interpretation limits its widespread use [23,24,25,26,27].

To overcome these limitations, the Pressure-Less Acoustic Immittance (PLAI™) system has been introduced as a truly non-invasive alternative. Based on microelectromechanical systems (MEMS), PLAI™ eliminates the need for ear canal pressurization while enabling multi-parameter measurements of middle ear function [28]. Although promising, PLAI™ relies on a novel paradigm and must therefore be validated against gold standard methods through rigorous clinical evaluation [29].

Initial investigations into PLAI™ suggest that its derived parameters exhibit strong physiological correlation and are sensitive to both pathology and patient age [30,31]. Therefore, the aim of this study is to determine whether age-dependent stratification improves diagnostic performance and whether non-invasive machine-learning based identification of common otologic pathologies can be achieved using PLAI™-derived features without reliance on conventional tympanometry.

## 2. Materials and Methods

### 2.1. Study Population

Outpatients aged from 0 to 80 years, both healthy and affected by various ear pathologies, were enrolled in the study from six Italian hospitals: Gemelli University Hospital IRCCS (Rome), “Federico II” University Hospital (Naples), Fondazione IRCCS Policlinico “San Matteo” Hospital (Pavia), “Giovanni XXIII” Children’s Hospital (Bari), “Guglielmo da Saliceto” Hospital (Piacenza), and IRCCS Maternal and Child Health Institute “Burlo Garofolo” (Trieste). Each patient underwent a clinical otologic examination, which included otoscopy and, where feasible, tympanometry to confirm the diagnosis. Following diagnosis, PLAI measurements were acquired to assess the middle ear’s acoustic properties under pressure-less conditions. The study was approved by the Ethics Committees of all participating institutions (Protocol No. 5389, 19 January 2023, IRCCS “A. Gemelli” Hospital, Rome), and by the Italian Ministry of Health (MEDWAVE-2, IT-23-03-042692, 9 May 2023). Written informed consent was obtained from adult participants; for minors, consent was provided by a parent or legal guardian.

Only patients with ears evaluated as healthy or as being affected by a single pathology were enrolled in the study, also excluding patients with a history of otologic surgery, significant comorbidities, and/or cognitive impairments that could affect measurements. All subjects underwent otoscopy and tympanometry to confirm the status of their auditory organs. Table 1 reports the distribution of individual ears by age group and diagnosis.

### 2.2. PLAI™ Device Description

**Figure 1:**
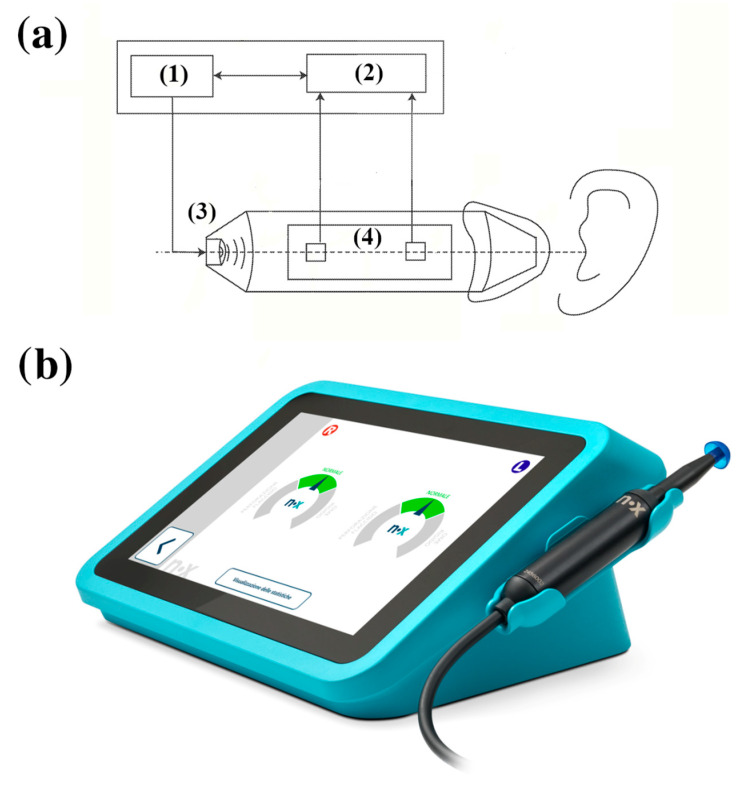
(**a**) Schematic of the Pressure-Less Acoustic Immittance (PLAITM) device, showing key components: (1) Sound source, (3) Speaker, (4) Sensors to capture reflected sound, and (2) Processing unit for data analysis. (**b**) Image of the PLAI^TM^ device.

The Pressure-Less Acoustic Immittance (PLAI™; Neuranix Srl, Naples, Italy) system is a MEMS-based diagnostic tool designed for middle ear assessment without the need for external pressurization. Unlike conventional tympanometry, PLAI operates under ambient pressure, using a dual-sensor setup capable of measuring both acoustic pressure and particle velocity at the same location (Figure 1). The device emits a wideband acoustic signal spanning 100 to 2000 Hz, which interacts with the ear canal and tympanic membrane. The reflected signal, containing key information about the mechanical and acoustic properties of the middle and outer ear, is captured by two microphones. These microphones measure the pressure and axial velocity of the air particles in the ear canal.

From the acquired signals, PLAI™ computes an acoustic admittance curve, from which it extracts numerous parameters, including the following:

- Resonance frequency (Fres);
- Peak admittance (Peak);
- Minimum and maximum frequency bounds (minBnd, maxBnd);
- Bandwidth amplitude (AltBnd);
- Equivalent ear canal volume (Vol).

The full feature set also includes age and labels used for classification.

### 2.3. Statistical Analysis

To assess whether PLAI™-derived parameters differ significantly between healthy and pathological ears, each acoustic feature was compared across diagnosis groups within each age category using Wilcoxon rank-sum test (Bonferroni correction was applied for multiple comparisons). A significance threshold of *p* < 0.05 was set.

### 2.4. Classification Pipeline

Following statistical analysis, a classification pipeline was implemented to evaluate the ability of PLAI™ features to distinguish between healthy and pathological ears.

The dataset was split into training (80%) and testing (20%) subsets within each age group. Feature selection was performed using a Random Forest classifier’s intrinsic feature importance score, retaining only the most relevant features per group. SMOTE (Synthetic Minority Over-sampling Technique) was applied to the training set to mitigate class imbalance by generating synthetic samples for underrepresented diagnostic categories.

Random Forest was selected as the classifier due to its robustness to overfitting, ability to handle nonlinear relationships, and compatibility with feature importance analysis. Hyperparameters were tuned based on performance on the validation split. Model evaluation included accuracy, precision, recall, F1-score, and specificity, computed both per class and as micro- and macro-averages. ROC curves were plotted, and confusion matrices were generated in grayscale for each age category.

To support model interpretability, SHAP (SHapley Additive exPlanations) values were computed, revealing the impact of individual features on classification decisions [32]. Feature importance visualizations are included in the results section for each age group.

All analyses and visualizations were performed in Python 3.8 using Scikit-learn, Imbalanced-learn, and SHAP libraries.

## 3. Results

### 3.1. Descriptive Statistics

#### 3.1.1. Age 0–3

In Table 2, we report the descriptive statistics and comparative analysis of key PLAI™ parameters between healthy children and those diagnosed with Otitis Media with Effusion (OME) in the 0–3 year age group. Statistically significant differences were found in multiple parameters.

**Figure 2:**
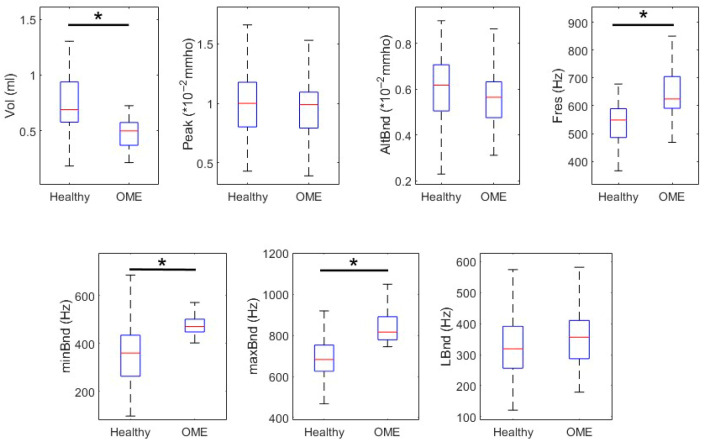
Box plots comparing PLAI parameters between Healthy and OME subjects in the 0–3 age group. Statistically significant differences are indicated by asterisks.

These findings are visually represented in Figure 2 through comparative boxplots, highlighting the dispersion and central tendency of each parameter.

#### 3.1.2. Age 3–12

**Figure 3:**
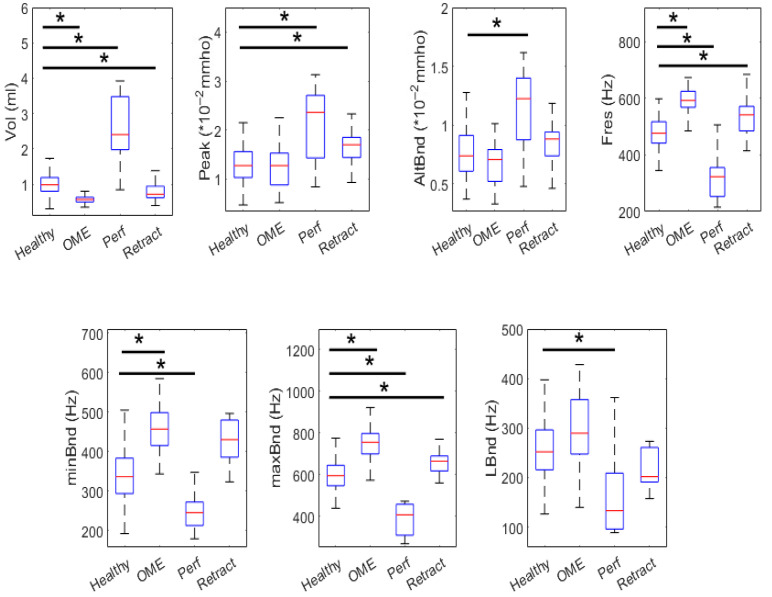
Box plots comparing PLAI parameters between Healthy, OME, Perforated, and Retracted ears in the 3–12 age group. Statistically significant differences are indicated by asterisks.

In Table 3, we report the descriptive statistics and comparative analysis for the main PLAI™-derived parameters in children aged 3 to 12 years, divided into healthy individuals, those diagnosed with Otitis Media with Effusion (OME), and individuals with tympanic membrane retraction. The comparisons were performed using appropriate statistical tests, with a Bonferroni-corrected significance threshold of *p* < 0.0167. These findings can be observed in boxplot form in Figure 3.

#### 3.1.3. Age Greater than 12

**Figure 4:**
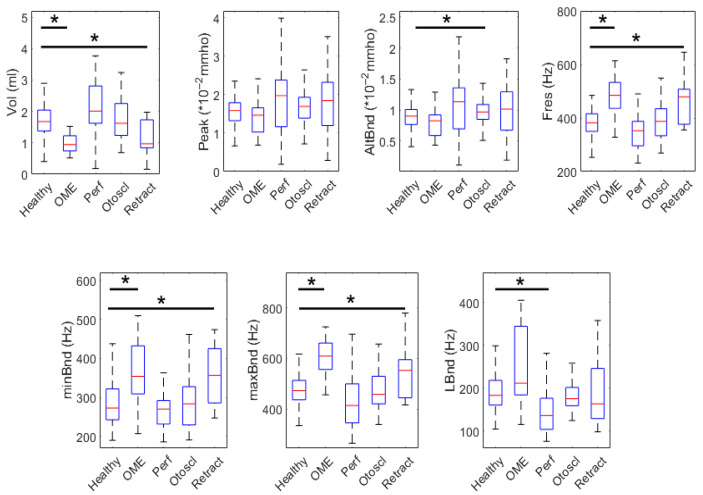
Box plots comparing PLAI parameters between Healthy, OME, Perforated, Otosclerotic, and Retracted ears in the 12+ age group. Statistically significant differences are indicated by asterisks.

In Table 4 we present the descriptive statistics and group comparisons for each parameter in the age group older than 12 years. This table includes the mean, median, standard deviation (SD), first (25th percentile) and third (75th percentile) quartiles, along with *p*-values comparing the healthy group with each pathological condition. The comparison threshold is set to *p* < 0.0125 to account for multiple comparisons. The corresponding boxplots are shown in Figure 4. Notably, there are no statistically significant differences in any of the parameters for otosclerosis in this age group, suggesting that the parameters analyzed may have limited discriminative value for this condition within the current feature set.

### 3.2. Feature Relevance via SHAP Analysis

To interpret the relevance of different features in the classification process, SHAP (SHapley Additive exPlanations) analysis was performed for each age group. Table 5 presents the mean SHAP values for the top-ranked features selected by the Random Forest classifier across the three age strata. These values quantify the average contribution of each feature to the model’s output.

For the 0–3 age group, the most influential feature was Fres, followed by maxBnd, minBnd, and Vol, suggesting that the frequency-related parameters played a central role in the classification of middle ear conditions. In the 3–12 age group, Vol and Fres again emerged as prominent contributors, alongside minBnd and LBnd, pointing to consistent diagnostic value across developmental stages. In adolescents and adults (12+), Vol, Fres, and AltBnd were key contributors, indicating that the volumetric and resonance features maintain importance in older patients as well.

These findings underscore the physiological plausibility of the selected features, as tympanic volume and resonance boundaries are commonly affected by pathological middle ear conditions such as effusions, retractions, or perforations.

### 3.3. Random Forest Classification Performance

Random Forest classifiers were trained and evaluated for each age group using a 5-class diagnostic classification task. Synthetic oversampling Via SMOTE [33] was employed to mitigate class imbalance. Performance was assessed using macro-averaged and micro-averaged metrics for precision, recall, F1-score, and specificity. Additionally, class-wise accuracy was extracted from the classification reports.

**Figure 5:**
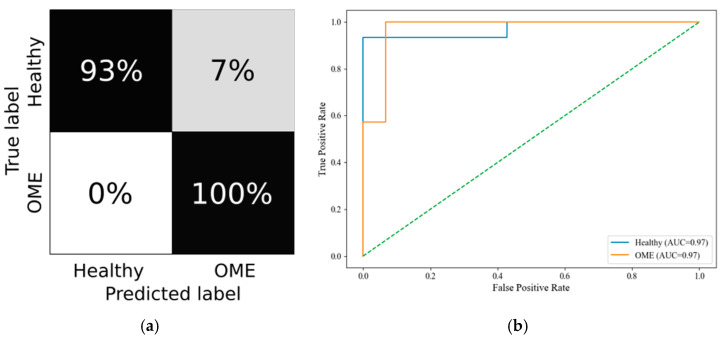
The 0–3 group RF metrics. (**a**) Confusion Matrix; (**b**) ROC Curve.

The 0–3 year group yielded a macro F1-score of 0.79 and a micro F1-score of 0.80, indicating good overall performance despite developmental anatomical variability. The model maintained a macro specificity of 0.95, emphasizing its reliability in distinguishing between pathological and non-pathological cases. The confusion matrix and ROC curves are depicted in Figure 5a and Figure 5b, respectively.

**Figure 6:**
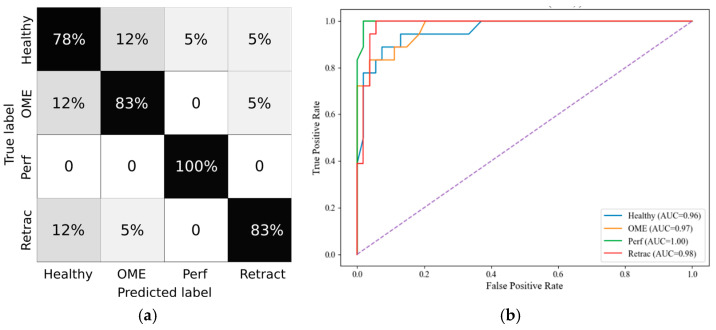
The 3–12 group RF metrics (**a**) Confusion Matrix; (**b**) ROC Curve.

In the 3–12 group, the classifier demonstrated the highest performance, with a macro F1-score of 0.84 and a micro F1-score of 0.86. Notably, the macro precision (0.85) and specificity (0.96) affirm strong discriminatory capacity in this age range, which typically presents frequent but well-characterized middle ear pathologies. The confusion matrix and ROC curves are depicted in Figure 6a and Figure 6b, respectively.

**Figure 7:**
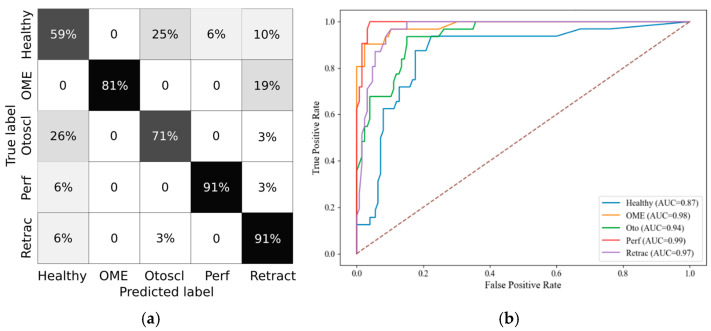
The 12+ group RF metrics. (**a**) Confusion Matrix; (**b**) ROC Curve.

In the 12+ group, performance remained robust, albeit slightly reduced, with a macro F1-score of 0.78 and micro F1-score of 0.80. The confusion matrix and ROC curves are depicted in Figure 7a and Figure 7b, respectively. This may reflect increased inter-individual anatomical variability and the presence of more diverse pathophysiological mechanisms in adolescents and adults.

The overall classification performance is summarized in Table 6, while the per-class classification accuracy is reported in Table 7.

## 4. Discussion

This study investigated whether stratifying pediatric subjects by age groups improves the diagnostic modeling of middle ear conditions using quantitative PLAI™ features. Motivated by the known developmental changes in the auditory system during infancy and childhood [34,35], we hypothesized that age-related physiological differences significantly influence tympanometric measurements, thus impacting classification accuracy when predicting pathologies such as otitis media with effusion (OME), tympanic membrane perforation (Perf), otosclerosis (Otoscl), and retraction (Retract).

The primary aim of this study was to determine whether stratifying by age reveals statistically significant differences between diagnostic groups and to evaluate the performance of machine learning classifiers trained within these age-defined strata for detecting ear pathologies without relying on conventional, and potentially invasive, tympanometry.

Statistical comparisons of wideband tympanometry parameters showed that age stratification enhances the discrimination between healthy and pathological ears, particularly in the youngest (0–3 years) and oldest (12+ years) age groups. These results justified the creation of age-specific models, which subsequently demonstrated robust classification performance. Specifically, random forest classifiers, trained with SMOTE-balanced datasets, achieved macro F1-scores above 80% in both the 0–3 and 12+ groups. The SHAP analysis further revealed consistent feature relevance patterns, with Peak, Fres, and Vol emerging as the most discriminative parameters across all age groups.

Importantly, stratifying the analysis by age groups led to a marked reduction in false negative rates for conditions like OME and tympanic membrane retractions. This improvement strengthens diagnostic reliability and highlights the model’s potential utility as a non-invasive screening tool in clinical settings. In particular, the PLAI™ system could be integrated into routine examinations by general practitioners and pediatricians, enabling them to assess ear health rapidly and with minimal training. By flagging abnormal patterns suggestive of middle ear pathology, the system could support early identification and prompt referral of at-risk children for further evaluation by an otolaryngologist. This triage function may be especially valuable in primary care and community health settings where access to specialist equipment and expertise is limited.

Physiologically, these observations align with known developmental changes in the ear canal and middle ear structures. In infants and toddlers (0–3 years), the ear canal is shorter and more compliant, while the ossicular chain and tympanic membrane undergo maturation that can dramatically affect acoustic transfer functions [36]. This likely contributes to the distinct parameter distributions observed in this group and underscores the need for specialized diagnostic thresholds in early life. Conversely, adolescents (12+ years) display more adult-like anatomy and auditory responses, which could explain the enhanced classification performance and clearer parameter separation in this group.

Among the tympanometric features, Peak admittance, representing energy flow at the resonant frequency, was repeatedly selected as a key predictor. Fres, the resonant frequency of the middle ear system, also showed high discriminative power, likely reflecting changes in ossicular ant tympanic stiffness. Volume (Vol), while highly informative in distinguishing perforations, showed some limitations in overlapping pathologies such as OME and retractions, particularly in the middle group (3–12 years), where developmental heterogeneity may blur diagnostic distinctions.

Despite promising results, this study has limitations. The dataset, while substantial, exhibited class imbalance, particularly in less frequent pathologies such as otosclerosis and retraction, which may affect classifier generalizability. While SMOTE was employed to address class imbalance, particularly for underrepresented diagnoses such as otosclerosis and tympanic membrane retraction, several limitations should be considered. SMOTE synthesizes new examples by interpolating between existing minority class samples, which may not fully capture the variability inherent in complex or rare pathologies. Moreover, age bins were defined in three broad categories; finer stratification might yield further insights but would require a larger cohort to maintain statistical power. In this study, the small samples available for these rare conditions were assumed to be representative of their respective classes. However, this assumption carries inherent risks: if the original samples do not adequately reflect the broader clinical variability of the condition, the synthetic data may reinforce sampling biases rather than mitigate them. Future work should prioritize collecting more real-world data for the minority classes to improve generalizability and reduce reliance on synthetic augmentation.

Lastly, although the models demonstrated strong diagnostic potential, especially in early and late childhood, further validation on external datasets is necessary to confirm generalizability. Expanding the dataset, especially with more balanced class representation and longitudinal follow-ups, would be beneficial.

Nonetheless, the study underscores the importance of age-aware diagnostic modeling in pediatric audiology and highlights PLAI™ as a promising non-invasive tool for early and reliable identification of middle ear conditions.

## 5. Conclusions

This study demonstrated that age-stratified analysis enables the classification of common otologic pathologies using a non-invasive screening methodology. By dividing subjects into physiologically meaningful age groups (0–3, 3–12, and 12+ years), we observed enhanced parameter discrimination and superior classification performance, particularly in the youngest and oldest cohorts. The application of random forest classifiers, combined with SHAP-based feature relevance analysis, identified Peak admittance, Fres, and Volume as key contributors to diagnostic accuracy.

These findings highlight the critical role of age-dependent anatomical and physiological changes in shaping acoustic immittance responses, reinforcing the necessity of age-specific diagnostic frameworks. Despite limitations related to class imbalance and sample size, our results indicate strong potential for integrating machine learning and tympanometric features into future clinical tools for early detection of middle ear pathologies.

Further research is warranted to validate these models in larger, more diverse populations and to refine age-specific diagnostic thresholds. Future work will also aim to establish diagnostic criteria for specific ear pathologies based on PLAI™ measurements and to validate these criteria through comparison with established clinical gold standards, such as otoscopic evaluation and audiological testing. Ultimately, this approach holds promise for improving diagnostic precision and guiding early interventions in pediatric audiology.

## Figures and Tables

**Table 1:** Distribution of individual ears by age and pathology.

Age Group	Healthy	OME	Perforation	Otosclerosis	Retraction	Total
0–3 years	71	22	-	-	-	**93**
3–12 years	90	28	7	-	18	**143**
12+ years	157	18	18	63	17	**273**
Total	318	68	25	63	35	**509**

**Table 2:** Descriptive statistics and *p*-values for PLAI™ parameters in the 0–3 year age group (*p*-values in bold are statistically significant).

Parameter	Group	Mean	SD	*p*-Value
**Volume (mL)**	Healthy	0.78	0.333	**<0.001**
	OME	0.62	0.666	
**Peak (×10^−2^ mmho)**	Healthy	1.02	0.353	0.732
	OME	1.01	0.398	
**AltBnd (×10^−2^ mmho)**	Healthy	0.61	0.19	0.184
	OME	0.58	0.19	
**Fres (Hz)**	Healthy	548	94	**<0.001**
	OME	638	132	
**minBnd (Hz)**	Healthy	357	118	**<0.001**
	OME	470	90	
**maxBnd (Hz)**	Healthy	711	151	**<0.001**
	OME	827	154	
**LBnd (Hz)**	Healthy	354	177	0.193
	OME	356	123	

**Table 3:** Descriptive statistics and *p*-values for PLAI™ parameters in the 3–12 year age group (*p*-values in bold are statistically significant).

Parameter	Group	Mean	SD	*p*-Value		
				OME	Perforation	Retraction
**Volume (mL)**	Healthy	1.06	0.40	**<0.001**	**<0.001**	**0.002**
	OME	0.59	0.17			
	Perforation	2.58	1.07			
	Retraction	0.81	0.29			
**Peak (×10^−2^ mmho)**	Healthy	1.29	0.37	0.740	**0.0103**	**0.005**
	OME	1.27	0.44			
	Perforation	2.12	0.83			
	Retraction	1.59	0.45			
**AltBnd (×10^−2^ mmho)**	Healthy	0.76	0.20	0.128	**0.010**	0.12
	OME	0.69	0.21			
	Perforation	1.13	0.39			
	Retraction	0.83	0.21			
**Fres (Hz)**	Healthy	477	65	**<0.001**	**<0.001**	**0.002**
	OME	596	68			
	Perforation	323	96			
	Retraction	531	69			
**minBnd (Hz)**	Healthy	336	77	**<0.001**	**0.005**	0.105
	OME	453	71			
	Perforation	248	54			
	Retraction	427	58			
**maxBnd (Hz)**	Healthy	595	82	**<0.001**	**0.002**	**0.003**
	OME	754	96			
	Perforation	414	148			
	Retraction	656	83			
**LBnd (Hz)**	Healthy	259	74	0.022	**0.006**	0.058
	OME	301	90			
	Perforation	167	98			
	Retraction	229	69			

**Table 4:** Descriptive statistics and *p*-values for PLAI™ parameters in the 12+ year age group (*p*-values in bold are statistically significant).

Parameter	Group	Mean	SD	*p*-Value			
				OME	Perf	+	Retract
**Volume (mL)**	Healthy	1.76	0.55	**<0.001**	0.101	0.929	**<0.001**
	OME	1.04	0.44				
	Perforation	2.06	0.97				
	Otosclerosis	1.78	0.66				
	Retraction	1.15	0.54				
**Peak (×10^−2^ mmho)**	Healthy	1.57	0.39	0.245	0.095	0.094	0.100
	OME	1.53	0.69				
	Perforation	1.96	1.06				
	Otosclerosis	1.66	0.38				
	Retraction	1.83	0.81				
**AltBnd (×10^−2^ mmho)**	Healthy	0.90	0.21	0.104	0.052	0.014	0.091
	OME	0.85	0.35				
	Perforation	1.12	0.55				
	Otosclerosis	0.96	0.18				
	Retraction	1.04	0.44				
**Fres (Hz)**	Healthy	384	58	**<0.001**	0.101	0.929	**<0.001**
	OME	484	75				
	Perforation	388	161				
	Otosclerosis	386	67				
	Retraction	489	152				
**minBnd (Hz)**	Healthy	284	56	**<0.001**	0.502	0.761	**<0.001**
	OME	366	81				
	Perforation	302	146				
	Otosclerosis	286	69				
	Retraction	380	143				
**maxBnd (Hz)**	Healthy	477	73	**<0.001**	0.039	0.670	**0.003**
	OME	612	70				
	Perforation	463	177				
	Otosclerosis	473	70				
	Retraction	573	172				
**LBnd (Hz)**	Healthy	193	56	0.013	**0.002**	0.308	0.389
	OME	246	92				
	Perforation	161	87				
	Otosclerosis	187	48				
	Retraction	193	81				

**Table 5:** Mean SHAP values for PLAI™ derived features.

Feature	0–3 Years	3–12 Years	12+ Years
Vol	0.1461	0.0908	0.1573
Peak	0.0306	0.0374	0.0343
AltBnd	0.0572	0.0484	0.0739
Fres	0.1603	0.0858	0.1242
minBnd	0.1110	0.0739	0.0516
maxBnd	0.1461	0.0380	0.0529
LBnd	0.0967	0.0696	0.0384

**Table 6:** Classification performance metrics of Random Forest classifiers for each age group.

Metric	0–3 Years	3–12 Years	12+ Years
Macro Precision	0.80	0.85	0.77
Micro Precision	0.81	0.86	0.80
Macro Recall	0.78	0.84	0.79
Micro Recall	0.81	0.86	0.80
Macro F1-score	0.79	0.84	0.78
Micro F1-score	0.81	0.86	0.80
Macro Specificity	0.95	0.96	0.93

**Table 7:** Per class accuracy of obtained classifiers.

Per Class Accuracy	0–3 Years	3–12 Years	12+ Years
Healthy	0.93	0.78	0.59
OME	1.0	0.83	0.81
Otosclerosis	-	-	0.71
Perforation	-	1.0	0.90
Retraction	-	0.83	0.90

## Data Availability

The datasets presented in this article are not readily available because the data are part of an ongoing study. Requests to access the datasets should be directed to Neuranix Srl.

## Abbreviations

The following abbreviations are used in this manuscript:

OME	Otitis Media with Effusion
PLAI	Pressure-Less Acoustic Immittance
SHAP	SHapley Additive exPlanations
RF	Random Forest
